# Efficacy of fenofibrate for diabetic retinopathy: A systematic review protocol

**DOI:** 10.1097/MD.0000000000014999

**Published:** 2019-04-05

**Authors:** Xing-jie Su, Lin Han, Yan-Xiu Qi, Hong-wei Liu

**Affiliations:** a Department of Ophthalmology, First Affiliated Hospital of Jiamusi University; b Department of Otorhinolaryngology, The 163th Hospital of the People's Liberation Army Joint Service Support Force, Jiamusi, China.

**Keywords:** diabetic retinopathy, efficacy, fenofibrate, safety, systematic review

## Abstract

**Background:**

Numerous studies have reported the efficacy of fenofibrate for patients with diabetic retinopathy (DRP). No systematic review has, however, addressed its efficacy for DRP. Thus, this systematic review will firstly evaluate the efficacy and safety of fenofibrate for patients with DRP.

**Methods:**

This study will search the following databases: PUMBED, EMBASE, CINAHI, ACMD, CENTRAL, CBM, CNKI, VIP, and WANGFANG, along with grey literature from inception to the present. We will accept randomized controlled trials on evaluating the efficacy and safety of fenofibrate for DRP. The primary outcome is the progression of DRP. The secondary outcomes are vision loss, development of diabetic macular edema, aggravation of hard exudates, quality of life, and any adverse events. Methodological quality of each included study will be assessed by using Cochrane Collaboration risk of bias tool. In addition, Grading of Recommendations Assessment, Development and Evaluation tool will also be used to evaluate the overall strength of the evidence. Two independent reviewers will conduct all procedures of study selection, data extraction, and methodological assessment. Any disagreements will be consulted with a third reviewer. RevMan 5.3 software will be used to pool data and to carry out the meta-analysis if it is possible.

**Results:**

In present study, we anticipate to find a considerable number of published studies presenting evidence on efficacy and safety of fenofibrate for DRP.

**Conclusion:**

The findings of this systematic review will provide latest evidence of fenofibrate for patients with DRP.

**Dissemination and ethics:**

The findings of this scoping review will be disseminated in print, conferences, or by peer-reviewed journals. No ethical approval is needed for this systematic review, because it is a literature-based study.

**Systematic review registration:**

PROSPERO CRD42019121869.

## 1 Introduction

Diabetic retinopathy (DRP) is one of the most common microvascular complications among patients with diabetes.^[1–3]^ It has been reported that DRP is responsible for 4.8% of 37 million cases of blindness worldwide.^[4]^ Unfortunately, no symptoms can be detected in early stage of DRP.^[5–7]^ When the visual issues are identified, it has already developed as advanced stage with a point of no return.^[8,9]^

Currently, intravitreal injections of antivascular endothelial growth factor are accepted as standard management for DRP.^[10–13]^ It still, however, has limited efficacy for some patients. Thus, alternative therapies are still needed to be explored. Fenofibrate is reported to be an alternative intervention for DRP.^[14–16]^ Numerous clinical trials have reported that it has promising efficacy for patients with DRP.^[17–24]^ No systematic review has, however, addressed this issue. Therefore, this systematic review will assess the efficacy and safety of fenofibrate for patients with DRP.

## 2 Methods and analysis

### 2.1 Study registration

This systematic review has been registered on PROSPERO (CRD42019121869). It is reported following the Preferred Reporting Items for Systematic Reviews and Meta-analysis Protocol.^[25]^

### 2.2 Study selection criteria

#### 2.2.1 Type of studies

We will include randomized controlled trials (RCTs) of fenofibrate for DRP. Nonclinical trials, noncase control studies, non-RCTs, and quasi-RCTs will, however, be excluded.

#### 2.2.2 Type of participants

We will include any clinically diagnosed criteria of DRP regardless of race, sex, and age.

#### 2.2.3 Type of interventions

We will accept studies that have implemented fenofibrate alone as an experimental treatment in any forms. Control treatment can be any kinds of interventions, except fenofibrate.

#### 2.2.4 Types of outcomes

The study reporting at least one of the following outcomes will be included. The primary outcome includes the progression of DRP. The secondary outcomes consist of vision loss, development of diabetic macular edema, aggravation of hard exudates, quality of life, and any adverse events.

### 2.3 Identifying relevant studies

This systematic review will summarize evidence published by primary trials and grey literature. The following databases will be searched from the inception to the present: PUMBED, EMBASE, CINAHI, ACMD, CENTRAL, CBM, CNKI, VIP, and WANGFANG. In addition, grey literature, such as relevant articles from the reference lists, Web sites of clinical trial registry, and doctorial dissertation will also be searched. The detailed search strategy for CENTRAL is presented in Table 1. The identical search strategies will also be applied to other electronic databases.

**Table 1:**
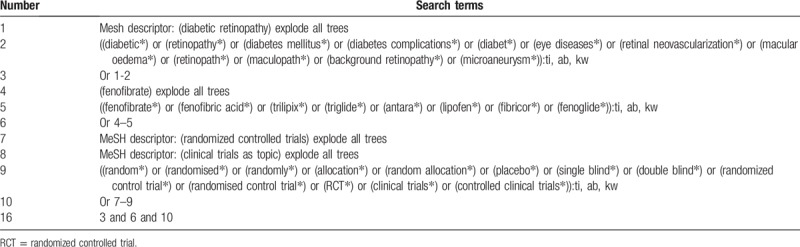
Search strategy applied in CENTRAL database.

### 2.4 Study selection

**Figure 1:**
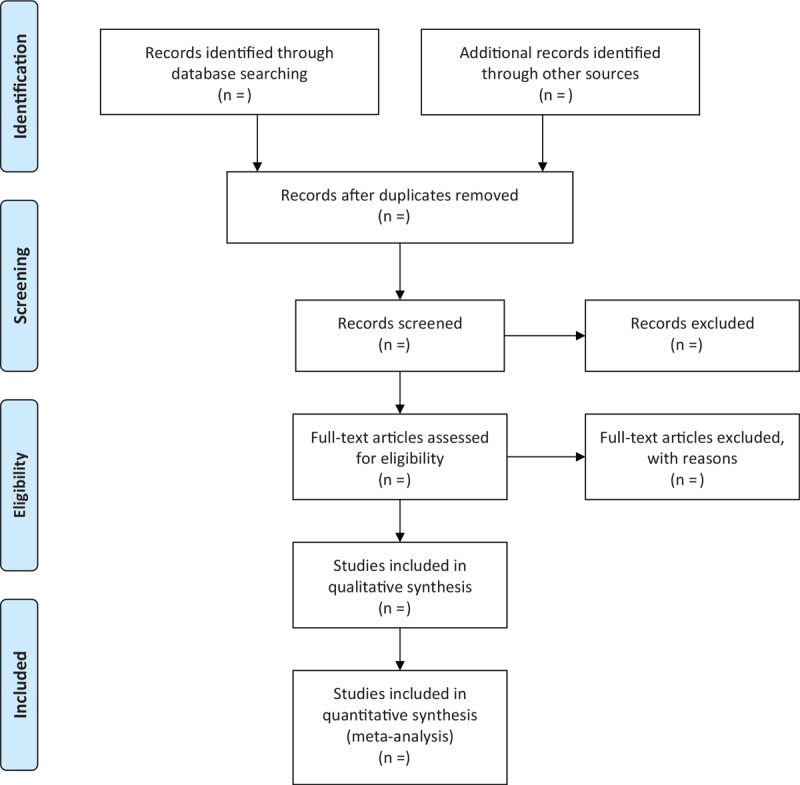
Flowchart of study selection.

Two reviewers will independently select the studies by scanning titles and summaries, and reading full-texts if it is necessary according to the predefined eligibility criteria. Any disagreements regarding the study selection will be solved by consulting a third reviewer through discussion. The whole process of study selection is summarized as flowchart in Figure 1.

### 2.5 Data extraction and management

Endnote 7 will be used to manage data by 2 independent reviewers. All data will be extracted according to the predefined standard data extraction sheet. It includes information of study characteristics (title, first author, year of publication, journal, country, and funding sources); patient characteristics (diagnosis criteria, comorbidities, race, sex, age); study method (sample size, randomization, concealment, blinding, and other potential risk bias); intervention details (type, dosage, frequency, and duration); and outcomes (primary, secondary, and other outcomes). Any discrepancies regarding the data extraction will be settled down by consulting a third author.

### 2.6 Dealing with missing data

If any data are missing, we will contract authors of primary studies. If we cannot achieve those data, then we will just analyze the available data and also discuss its potential impacts.

### 2.7 Risk of bias assessment

Two reviewers will independently assess the methodological quality for each included trial by using Cochrane Handbook for Systematic Reviews of Interventions Tool.^[26]^ Each item will be judged according to the standard criteria of Cochrane risk of bias tool.^[26]^ Any disagreements will be solved by consensus with a third author.

### 2.8 Quality of evidence rating

Grading of Recommendations Assessment, Development and Evaluation (GRADE) tool will be used to evaluate the overall strength of the evidence.^[27]^ Its results will be summarized in tables of Summary of Findings.

### 2.9 Statistical analysis

RevMan 5.3 software will be used to pool the data and to conduct the meta-analysis. All continuous data are expressed as mean difference or standardized mean difference with 95% confidence intervals (CIs). All dichotomous data will be expressed as risk ratio with 95% CIs.

Heterogeneity will be checked by *I*^2^ test. Acceptable heterogeneity will be considered if *I*^2^ ≤50, and a fixed-effect model will be used to synthesize the data. Otherwise, substantial heterogeneity will be considered, and a random-effect model will be used to synthesize the data. Under such situation, subgroup analysis will be carried out according to the different interventions, research scenario, and outcome tools. If significant heterogeneity is still identified after subgroup analysis, then data will not be recommended to synthesize, and meta-analysis will not be carried out. Instead, a narrative summary will be presented.

Whenever possible, sensitivity analysis will be performed to check the robustness of pooled results data by removing low quality of studies. In addition, Funnel plot and Egg's regression will be conducted if >10 eligible trials are included in this study.

## 3 Discussion

In this systematic review, we will evaluate the methodological quality by using Cochrane risk of bias tool and assess the quality of evidence with GRADE tool. Two independent reviewers will conduct the study selection, data extraction, and methodological quality assessment, whereas any disagreements will be settled down with a third reviewer through discussion. This study will generate present evidence of fenofibrate for patients with DRP, and will help to reduce the uncertainty about the efficacy and safety of fenofibrate management. The findings of this study will encourage further suggestions for clinicians or guideline, and will draw wide attention for both patients and researchers.

## Author contributions

**Conceptualization:** Xing-jie Su, Hong-wei Liu, Yan-Xiu Qi.

**Data curation:** Xing-jie Su, Lin Han, Hong-wei Liu.

**Formal analysis:** Xing-jie Su, Lin Han, Yan-Xiu Qi.

**Funding acquisition:** Hong-wei Liu.

**Investigation:** Hong-wei Liu.

**Methodology:** Xing-jie Su, Lin Han, Yan-Xiu Qi.

**Project administration:** Hong-wei Liu.

**Resources:** Xing-jie Su, Lin Han, Yan-Xiu Qi.

**Software:** Xing-jie Su, Lin Han, Yan-Xiu Qi.

**Supervision:** Hong-wei Liu.

**Validation:** Lin Han, Hong-wei Liu.

**Visualization:** Xing-jie Su, Yan-Xiu Qi.

**Writing - Original Draft:** Xing-jie Su, Lin Han, Hong-wei Liu.

**Writing - Review and Editing:** Xing-jie Su, Lin Han, Hong-wei Liu, Yan-Xiu Qi.

## References

[1] Kusuhara S Fukushima Y Ogura S Pathophysiology of diabetic retinopathy: the old and the new. Diabetes Metab J 2018;42:364–76. 30362302 10.4093/dmj.2018.0182 PMC6202564

[2] Mumtaz SN Fahim MF Arslan M Prevalence of diabetic retinopathy in Pakistan: a systematic review. Pak J Med Sci 2018;34:493–500. 29805433 10.12669/pjms.342.13819 PMC5954404

[3] Lu L Jiang Y Jaganathan R Current advances in pharmacotherapy and technology for diabetic retinopathy: a systematic review. J Ophthalmol 2018;2018:1694187. 29576875 10.1155/2018/1694187 PMC5822768

[4] Resnikoff S Pascolini D Etya’ale D Global data on visual impairment in the year 2002. Bull World Health Organ 2004;82:844–51. 15640920 PMC2623053

[5] Shah AR Gardner TW Diabetic retinopathy: research to clinical practice. Clin Diabetes Endocrinol 2017;3:9. 29075511 10.1186/s40842-017-0047-y PMC5648499

[6] Lynch SK Abràmoff MD Diabetic retinopathy is a neurodegenerative disorder. Vision Res 2017;139:101–7. 28408138 10.1016/j.visres.2017.03.003 PMC5659971

[7] Nørgaard MF Grauslund J Automated screening for diabetic retinopathy—a systematic review. Ophthalmic Res 2018;60:9–17. 29339646 10.1159/000486284

[8] Chua J Lim CXY Wong TY Diabetic retinopathy in the Asia-Pacific. Asia Pac J Ophthalmol (Phila) 2018;7:3–16. 29376231 10.22608/APO.2017511

[9] Scanlon PH Screening intervals for diabetic retinopathy and implications for care. Curr Diab Rep 2017;17:96. 28875458 10.1007/s11892-017-0928-6 PMC5585285

[10] Smith JM Steel DH Anti-vascular endothelial growth factor for prevention of postoperative vitreous cavity haemorrhage after vitrectomy for proliferative diabetic retinopathy. Cochrane Database Syst Rev 2015;8:CD008214. 10.1002/14651858.CD008214.pub3 PMC6599827 26250103

[11] Ojha S Balaji V Sadek B Beneficial effects of phytochemicals in diabetic retinopathy: experimental and clinical evidence. Eur Rev Med Pharmacol Sci 2017;21:2769–83. 28678306

[12] Li X Zarbin MA Bhagat N Anti-vascular endothelial growth factor injections: the new standard of care in proliferative diabetic retinopathy? Dev Ophthalmol 2017;60:131–42. 28427072 10.1159/000459699

[13] Bressler SB Beaulieu WT Glassman AR Factors associated with worsening proliferative diabetic retinopathy in eyes treated with panretinal photocoagulation or ranibizumab. Ophthalmology 2017;124:431–9. 28161147 10.1016/j.ophtha.2016.12.005 PMC6648671

[14] Wang N Zou C Zhao S Fenofibrate exerts protective effects in diabetic retinopathy via inhibition of the ANGPTL3 pathway. Invest Ophthalmol Vis Sci 2018;59:4210–7. 30128492 10.1167/iovs.18-24155

[15] Stewart S Lois N Fenofibrate for diabetic retinopathy. Asia Pac J Ophthalmol (Phila) 2018;7:422–6. 30058790 10.22608/APO.2018288

[16] Deng G Moran EP Cheng R Therapeutic effects of a novel agonist of peroxisome proliferator-activated receptor alpha for the treatment of diabetic retinopathy. Invest Ophthalmol Vis Sci 2017;58:5030–42. 28979999 10.1167/iovs.16-21402 PMC5633008

[17] Keech AC Mitchell P Summanen PA Effect of fenofibrate on the need for laser treatment for diabetic retinopathy (FIELD study): a randomised controlled trial. Lancet 2007;370:1687–97. 17988728 10.1016/S0140-6736(07)61607-9

[18] Simó R Hernández C Fenofibrate for diabetic retinopathy. Lancet 2007;370:1667–8. 17988727 10.1016/S0140-6736(07)61608-0

[19] Wen ZY Effect of fenofibrate on the treatment of diabetic retinopathy with nephropathy. Chin J Med Sci 2018;8:54–6.

[20] Du YP Liu Y Guan YH The efficacy of fenofibrate in the treatment of diabetic retinopathy with nephropathy. Chin J Endemiol Prev Treat 2018;33:82–4.

[21] Zhong Y Clinical efficacy of fenofibrate in the treatment of non-proliferative diabetic retinopathy. Mod Diagn Treat 2015;26:3421–2.

[22] Xiao HB Lu JL Shao Y Clinical study of fenofibrate in the treatment of diabetic retinopathy with nephropathy. New Progress Ophthalmol 2014;34:1132–6.

[23] Chen SF Clinical study of fenofibrate in diabetic macular edema. Nanchang University (Dissertation). 2014.

[24] Zhao T Xu Q Clinical study of fenofibrate in the treatment of diabetic retinopathy. Clin Med 2011;31:51–2.

[25] Shamseer L Moher D Clarke M Preferred reporting items for systematic review and meta-analysis protocols (PRISMA-P) 2015: elaboration and explanation. BMJ 2015;349:g7647. 10.1136/bmj.g7647 25555855

[26] Higgins JP Altman DG Gøtzsche PC The Cochrane Collaboration's tool for assessing risk of bias in randomised trials. BMJ 2011;343:1–9. 10.1136/bmj.d5928 PMC3196245 22008217

[27] Guyatt GH Oxman AD Vist GE GRADE: an emerging consensus on rating quality of evidence and strength of recommendations. BMJ 2008;336:924–6. 18436948 10.1136/bmj.39489.470347.AD PMC2335261

